# Mesna ameliorates acute lung injury induced by intestinal ischemia–reperfusion in rats

**DOI:** 10.1038/s41598-021-92653-7

**Published:** 2021-06-25

**Authors:** Samia Adel Abd El-Baset, Manal R. Abd El-haleem, Rehab S. Abdul-Maksoud, Asmaa A. A. Kattaia

**Affiliations:** 1 Department of Medical Histology and Cell Biology, Faculty of Medicine, Zagazig University, Zagazig, 44519 Asharquia Egypt; 2 Faculty of Dentistry, Egyptian Russian University, Badr City, Egypt; 3 Department of Biochemistry, Faculty of Medicine, Zagazig University, Zagazig, Egypt

**Keywords:** Cell adhesion, Cell death

## Abstract

The lung is severely affected by intestinal ischemia–reperfusion (I–R) injury. Mesna, a thiol compound, possess anti-inflammatory and antioxidant properties. We aimed in the present work to explore the potential beneficial effects of Mesna on the acute lung damage mediated by intestinal I–R in a rat model. Forty male adult albino rats were randomly separated into; control, intestinal I–R, Mesna I and Mesna II groups. Mesna was administered by intraperitoneal injection at a dose of 100 mg/kg, 60 min before ischemia (Mesna I) and after reperfusion (Mesna II). Arterial blood gases and total proteins in bronchoalveolar lavage (BAL) were measured. Lung tissue homogenates were utilized for biochemical assays of proinflammatory cytokines and oxidative stress markers. Lung specimens were managed for examination by light and electron microscopy. Our results revealed that Mesna attenuated the histopathological changes and apoptosis of the lung following intestinal I–R. Mesna also recovered systemic oxygenation. Mesna suppressed neutrophil infiltration (as endorsed by the reduction in MPO level), reduced ICAM-1 mRNA expression, inhibited NF-κB pathway and reduced the proinflammatory cytokines (TNF-α, IL-1β and IL-6) in the lung tissues. Mesna maintained the antioxidant profile as evidenced by the elevation of the tissue GPx and SOD and down-regulation of HSP70 immune-expressions. Accordingly, Mesna treatment can be a promising way to counteract remote injury of the lung resulted from intestinal I–R.

## Introduction

Intestinal ischemia–reperfusion (I–R) injury is fatal condition which causes elevated mortality rates between 60 and 80%^1^. It presents a fundamental part in the pathophysiology of various clinical surgeries like acute mesenteric arterial occlusion, intestinal intussusception and hemodynamic shock^2^. It might be a complication of cardiopulmonary bypass^3^, aneurysm of abdominal aorta, hernia strangulation, necrotizing enterocolitis of neonates, transplantation of intestine^4^ and septic shock^5^.

Intestinal I–R injury causes interruption of the mucosal barrier of the intestine, resulting in severe generalised inflammation and consequent remote organ damage^6^. The lung is a highly susceptible affected organ by intestinal I–R^7^. Acute lung injury is a serious complication which could precede to death^8^.

Leukocyte-endothelial interaction plays a key role in lung damage through neutrophils stimulation to liberate inflammatory cytokines^9^. The nuclear factor kappa beta (NF-κB); a vital regulator of inflammation genes, can control these cytokines^10^.

Mesna (sodium-2- mercaptoethanesulfonate) is a thiol compound. Dimesna is formed by its metabolism. The action of glutathione dehydrogenase on the reabsorbed portion generates free sulfhydryl groups. Its excretion occurs through the kidneys^11^. It is extensively utilized as a mucolytic factor in respiratory disorders^12^, a chemical dissector in otologic and neurologic operations^13^ and as a preventive factor for hemorrhagic cystitis^14^. Also, Mesna is used to treat nephrotoxicity and urinary tract toxicity resulted from cyclophosphamide e.g. ifosfamide^15^. It is given in conjunction with cisplatin chemotherapy^16^.

Mesna is capable of scavenging reactive oxygen species (ROS)^17^. So, it could minimize ROS-induced tissue toxicity in various organs like small intestine^18^, liver^19^, colon^20^, ovary^21^, pancreas^22^ and lung^23^. The evident rise in surgical interferences and organ transplantations together with the increased morbidity and mortality rates resulting from ischemia–reperfusion disorders, have raised the interest in such disorders. Until now, no satisfactory measures proved to be successful in preventing or treating I–R injury. So, in the current work, our target was to study the potential beneficial effects of Mesna on the acute lung damage mediated by experimentally induced intestinal I–R in rats.

## Materials and methods

### Chemicals

Mesna (sodium 2-mercaptoethanesulfonate; Uromitexan; CAS No. 19767-45-4; purity of 98%; solid; water soluble) was stored at room temperature and obtained from Sigma-Aldrich (St. Louis, MO, USA).

### Experimental animals

Forty albino rats (male, weighing 200 to 250 g, aged 7 to 8 weeks). They were obtained and retained at the Breading Animal House, Faculty of Medicine, Zagazig University. Rats were allowed free access to food and water. Plastic cages with filter tops were used to house rats. They were held in controlled room, without any chemical pollution. The room was illuminated artificially (12 h dark:12 h) with a temperature of 23 ± 1 °C and a humidity of 55 ± 5%. They were kept for one-week acclimatization period. All experimental protocols were approved by the Medical Research Ethics Committee of Zagazig University, Egypt (The protocol approval number was 6734). The study was carried out in compliance with ARRIVE guidelines and the National Institutes of Health guide for the use and care of Laboratory animals.

### Experimental design

Rats were randomly separated to four groups (10 rats each). Control group (sham-operated); the superior mesenteric artery (SMA) was isolated without occlusion. Intestinal ischemia–reperfusion (I–R) group had been exposed to intestinal ischemia through clamping of the superior mesenteric artery for 1 h then reperfusion by declamping for 2 h^24^. The operation would be described below. Mesna I group was given intraperitoneal Mesna (at a dose of 100 mg/kg, dissolved in saline, 60 min before ischemia) then underwent surgery as intestinal I–R group. Mesna II group was given Mesna at the same dose after reperfusion. The Mesna dose was chosen depending on the literature^18^.

### Surgical technique

Rats were fastened overnight with free approach to water, then they received anesthesia by injection of ketamine (50 mg/kg) and xylazine (10 mg/kg) intramuscularly. A heating mat was used to preserve their body temperature (36 ± 1 ºC). The rats were put supine, then shaving of the abdomen and cleaning with antiseptic solution (povidone iodine 10%) were done. Abdominal incision (2–3 cm) was made in the mid-line, followed by exposure of the aorta and other visceral arteries.

In Sham group, SMA mobilization done without clamping. The incision was closed, followed by 3 h interval to simulate the I–R of the two other groups. In intestinal I–R, Mesna I and Mesna II groups, microvascular clamp (atraumatic) was positioned across the SMA at its beginning from the aorta, with caution to avoid superior mesenteric vein occlusion. Ischemia had been confirmed by observing intestinal paleness and absence of mesenteric pulse. After ischemia for 60 min, the microvascular clamp was withdrawn to allow reperfusion for 2 h. Reperfusion was indicated by return of intestinal color and pulsation. Throughout the surgical procedure, the entire bowel was protected with sterile pads soaked in saline at 37  °C to diminish evaporation and heat loss^25^.

By the operation end, animals were sacrificed. Median sternotomy was performed and the lungs were extracted from the thoracic cage. Tissue homogenates for biochemical assays were prepared, freezing of the right lungs was done immediately in liquid nitrogen, then storage at − 80 °C. Specimens from left lungs were managed for examination by light and electron microscopy.

### Arterial blood gases

Before scarification, 0.5 ml arterial blood was sampled from the abdominal aorta with a heparinized syringe. For measurements of partial pressure of O_2_ (PaO_2_), partial pressure of CO_2_ (PaCO_2_) and pH, an automated analyzer (AVL-Compact3, Roche Diagnostic, Germany) was used.

### Biochemical and molecular study

#### Proteins in bronchoalveolar lavage (BAL)

After scarification, exposure of the trachea and clamping of the left main bronchus were done. A catheter was placed into the trachea, through which 8 ml sterile cold saline was instilled. The lavage fluid was gently rinsed in and out 3 times before collection. Recovered fluids were centrifuged at 400×*g* for 10 min at 4 ºC. The supernatants were separated in ice-cold tubes and stored at –80 ºC till used. Measurement of the total proteins in BAL was achieved by BCA protein assay kits (Bio-Rad, California, USA) at absorbance of 750 nm.

#### Lung proinflammatory cytokines

The tissues of the lung were homogenized directly on ice in five volumes of normal saline. We used centrifugation of the homogenates at 1200×*g* for 10 min. We measured the levels of tumor necrosis factor-alpha (TNF-α), interleukin-6 (IL-6) and interleukin-1 beta (IL-1β) in the supernatant using kits of enzyme-linked immunosorbent assay (ELISA, Sigma-Aldrich, Street Louis, MO, USA) as stated in the manufacturer’s protocol.

#### Lung oxidative stress markers

##### Myeloperoxidase (MPO)

MPO was assessed to detect neutrophil sequestration. The activity of MPO was assayed using the modified method of Goldblum et al.^26^. Using hexadecyltrimethylammonium bromide buffer (HTAB) (phosphate buffer 50 mM having HTAB 0.5% at pH 6.0), frozen lung tissues were homogenized, then ultrasonicated and centrifuged for 15 min at 40,000×*g*. The activity of MPO was detected by measuring the *o*-dianisidinehydrochloride oxidation by H_2_O_2_ at 460 nm. The results were presented as units per gram protein.

##### Glutathione peroxidase (GPx)

Its activity was assessed by the GPx assay kit from Cayman chemicals (catalog #: 703102) following the manufacturer's protocol. One unit means the enzyme amount responsible for oxidation of 1.0 nmol of NADPH at 25 °C per minute. The GPx activity was presented as units per gram protein.

##### Total superoxide dismutase (T-SOD)

It was assessed using WST-1 Cell Proliferation Assay kit from ElabScience (catalog #: E-BC-K020-M) following the manufacturer's guidelines. One T-SOD unit represents the amount required for 50% of the superoxide radical dismutation. The activity of T-SOD was expressed as unit per gram protein.

#### Molecular study

##### Extraction of total RNA and reverse transcription polymerase chain reaction (RT-PCR) of intracellular adhesion molecule-1(ICAM-1)

Semi-quantitative RT-PCR was utilized following the method of Ji et al.^27^. TRIZOL (Invitrogen, USA) method was used to obtain total RNA from the frozen lung tissue following the protocol of the manufacturer. The kit of QuantiTect Reverse Transcription (Qiagen) was utilized for RNA reverse transcription. GAPDH (Glyceraldehyde-3-phosphate dehydrogenase) acted as a household control gene. PCR primers sequences (Invitrogen, Beijing, China) were as follow: ICAM-1 forward primer: 5’-CTTTGCCCTGGTCCTCCAAT-3’; reverse primer: 5’-TGTCTTCCCCAATGTCGCTC-3’ and GAPDH forward primer: 5’-TCC CTC AAG ATTGTC AGC AA-3’; reverse primer: 5’-AGA TCC ACA ACG GAT ACA TT-3’. PCR cycling conditions were: hot start at 94 °C for 3 min, then 30 cycles including 60 s at 94 °C, 90 s at 58 °C, and finally 3 min at 73 °C. We used agarose gel (1.5%) electrophoresis stained by ethidium bromide for assessment of PCR products. Gel documentation 1000 system (Bio-Rad, Munich, Germany) was used for densitometric test of the products. Relative expression of ICAM-1 was determined by dividing densitometric units of ICAM-1 by densitometric units of GAPDH.

### Histopathological study

#### Examination of light microscopy

##### Hematoxylin and eosin stain (H&E)

Buffered formalin (10%) was used as a fixative for light microscopy specimens which managed for the preparation of paraffin sections (5 μm-thick) to be stained by H&E^28^.

##### Immunohistochemical study

Localization of p38 mitogen-activated protein kinase (p38 MAPK), heat shock protein 70 (HSP70) and nuclear factor kappa beta (NF-κB), in the lung tissue by immunohistochemical staining was carried out. The method of avidin biotin–peroxidase complex was used following the manufacturer manual (Peroxidase, Dako ARK, Code No. K3954, Dako, Glostrup, Denmark).

Dewaxing and hydration of 5 μm-paraffin sections were performed. Antigens were retrieved by microwave treatment (0.01 M Trisodium citrate). Endogenous peroxidase was eradicated by using H_2_O_2_ (10%) in phosphate-buffered saline (PBS) at pH 7.4. At room temperature, tissues were blocked by a normal serum of mice. Incubation with the specific primary antibodies was done at 4 °C overnight. We tested anti-p38 MAPK (code No. ab31828; mouse monoclonal; 1:200 dilution; Abcam, Cambridge, UK), anti-HSP70 antibody (code No. ab5442; mouse monoclonal; 1/200 dilution; Abcam, Cambridge, UK) and anti-NF-κB (Cat. No. #RB-9034-R7; rabbit polyclonal; 1/100 dilution; Thermo Scientific, CA, USA). Secondary antibodies (biotinylated) and labelled horseradish peroxidase were added to sections. The chromogen, 3,3’-diaminobenzidine (DAB), was used to stain tissues at the antigen site forming in a brown color. Hematoxylin was used as counterstain. Negative controls were obtained by omitting the primary antibodies. Light microscopy was used for analysis of the stained slides^29^.

#### Examination of electron microscopy

Fixation of the specimens in phosphate-buffered glutaraldehyde (2.5%) at a pH of 7.4, then osmium tetroxide (1%) at 4 °C was the beginning points. Dehydration was done, followed by embedding in epoxy resin. Leica ultra-cut (UCT) was used for cutting sections which stained by lead citrate and uranyl acetate^30^. Examination and photography were performed by transmission electron microscopy (JEOL JEM 1010, JEOL Ltd, Tokyo, Japan) at Mycology and Biotechnology Center, Al- Azhar University, Egypt.

### Morphometric study

The image analyser computer system Leica QWin 500 (Leica Ltd, Cambridge, UK) was utilized for analysis of data. Olympus optical microscope (Tokyo, Japan) linked to a camera is connected to the software. The number of brown positive cells was evaluated in immune-stained sections of anti-p38 MAPK, anti HSP70 and anti-NF-κB. Inflammatory cell infiltrations in H&E stained sections were also counted. Using the interactive measure menu, all measurements were conducted at a 400 × magnification in frame area of 7286.78 µm^2^. From each animal in each group, the examiner chose randomly and analysed non-overlapping 10 fields. The analyses were done by an examiner who is blinded to the study.

### Statistical analysis

Data analyses were done by IBM SPSS software (version 23.0, IBM Corp, Armonk, NY, USA). Values were presented as mean ± standard error (X ± SE). The difference among groups was tested by ANOVA (one-way analysis of variance). Tukey’s HSD test (a post hoc test) was then done for the pairwise comparisons. When the probability values (*p*-values) were less than 0.05, they were considered significant. When they were less than 0.001, the were considered highly significant.

### Ethics approval and consent to participate

All rats provided with humane care in accordance with the guidelines of the Medical Research Ethics Committee of Zagazig University, Egypt and was conformed to the National Institutes of Health guide for the use and care of Laboratory animals.

## Results

### Arterial blood gases measurements

Intestinal I–R group showed significant decreases in PaO_2_ and pH and non-significant increase regarding PaCO_2_ when compared to other groups. Mesna administration significantly increased oxygenation and pH; Mesna I and Mesna II groups revealed non-significant differences when compared to the control (Table 1).

**Table 1:** Measurements of arterial blood gases and pH.

	Control group	Intestinal I–R group	Mesna I group	Mesna II group
PaO_2_ (mmHg)	98.17 ± 2.7	88.17 ± 1.2 ^#c^	96.33 ± 2.2^#o^	95.83 ± 0.9^#o^
PaCO_2_ (mmHg)	38.17 ± 0.5	39.17 ± 1.3	39.5 ± 1.4	38.5 ± 0.8
PH	7.41 ± 0.011	7.31.5 ± 0.009^##c^	7.38 ± 0.014^#o^	7.39 ± 0.010^##o^

Values are expressed as mean ± standard error (X ± SE).

PaO_2_, partial pressure of O_2_; PaCO_2_, partial pressure of CO_2_.

^#^Significant difference (*P* < 0.05).

^##^highly significant difference *(P* < 0.001); n = 6 animals.

^c^*P* compared to control group.

^o^*P* compared to intestinal I–R group.

### Biochemical and molecular results

#### Protein measurement in BAL

Analysis of the total proteins in intestinal I–R group revealed a highly significant increase in comparison with the control, Mesna I and Mesna II groups. However, there was non-significant difference between the controls and Mesna groups (Table 2).

**Table 2:** Biochemical parameters.

	Control group	Intestinal I–R group	Mesna I group	Mesna II group
Total proteins in BAL (μg/mL)	74.17 ± 4.3	157.33 ± 8.4^##c^	88.5 ± 7.9^##o^	82.17 ± 7.2^##o^
TNF-α (pg/mg protein)	15.9 ± 1.5	41.5 ± 2.9^##c^	19.1 ± 1.5^##o^	17.5 ± 1.6^##o^
IL-1β (pg/mg protein)	25.5 ± 1.9	65.4 ± 5.0^##c^	37.8 ± 5.7^#o^	32.4 ± 3.1^##o^
IL-6 (pg/mg protein)	21.1 ± 2.1	50.1 ± 4.3^##c^	28.4 ± 2.3^##o^	23.6 ± 2.7^##o^
MPO (U/g protein)	1.8 ± 0.2	12.4 ± 1.5^##c^	2.7 ± 0.4^##o^	3.5 ± 0.6^##o^
GPx (U/g protein)	38.6 ± 1.8	17.0 ± 1.6^##c^	30.9 ± 2.7^#c, ##o^	32.3 ± 2.3^##o^
T-SOD (U/g protein)	4.4 ± 0.3	1.9 ± 0.2^##c^	3.7 ± 0.4^#o^	3.9 ± 0.3^##o^

Values are expressed as mean ± standard error (X ± SE).

*BAL* bronchoalveolar lavage, *TNF-α* tumor necrosis factor-alpha, *IL-1β* interleukin-1 beta; IL-6, interleukin-6, *MPO* myeloperoxidase, *GPx* glutathione peroxidase, *T-SOD* total superoxide dismutase.

^#^Significant difference (*P* < 0.05).

^##^Highly significant difference *(P* < 0.001); n = 10 animals.

^c^*P* compared to control group.

^o^*P* compared to intestinal I–R group.

#### Lung proinflammatory cytokines

Measurements of the levels of lung proinflammatory mediators; TNF-α, IL-1β, and IL-6 showed significant increases in intestinal I–R group when compared to other groups. Mesna I and Mesna II groups revealed non-significant differences when compared to the control. The results were summarized in (Table 2).

#### Lung oxidative stress markers

Intestinal I–R group showed a significant elevation in tissue MPO level and a significant reduction in GPx and T-SOD in lungs in comparison to other groups. Mesna I group showed non-significant differences regarding MPO and T-SOD in comparison to controls. Mesna II group revealed non-significant differences regarding MPO, GPx and T-SOD when compared to Mena I and control groups.

#### Molecular results

**Figure 1:**
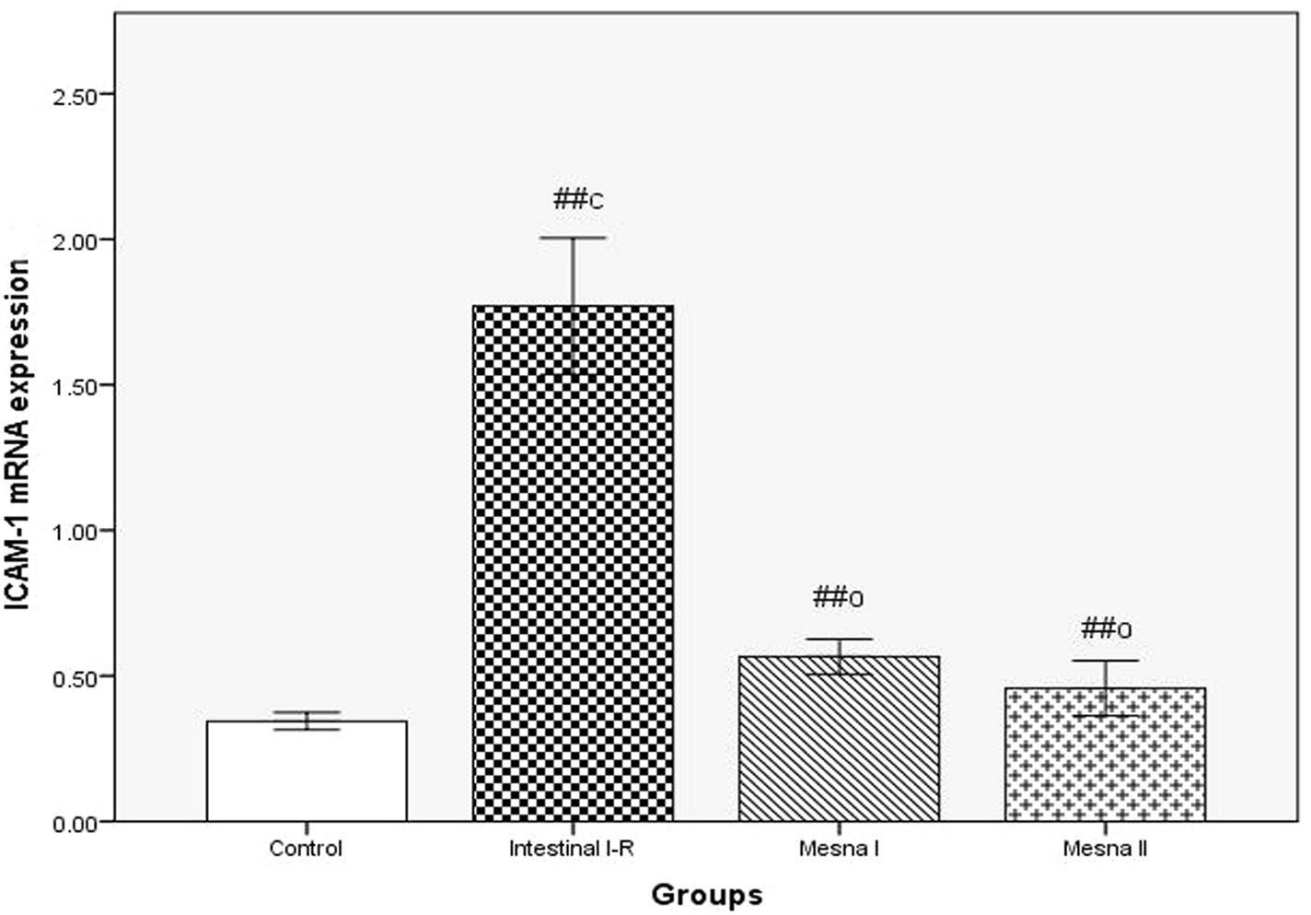
RT-PCR analysis of lungs' ICAM-1 mRNA expression estimated as ICAM-1/GAPDH ratio. Expression of values is as mean ± standard error (X ± SE); n = 10 animals; ^c^: *P* comparable to control group; ^o^: *P* comparable to intestinal I–R group; ^#^: *P* < 0.05; ^##^: *P* < 0.001.

A highly significant elevation in the expression of ICAM-1 mRNA was detected in intestinal I–R group in comparison to normal controls. Also, a highly significant reduction was shown in Mesna I and Mesna II groups when compared to intestinal I–R group and a non-significant difference in comparison to normal controls (Fig. 1).

### Histopathological results

#### Results of light microscopy

##### Results of H&E stain

**Figure 2:**
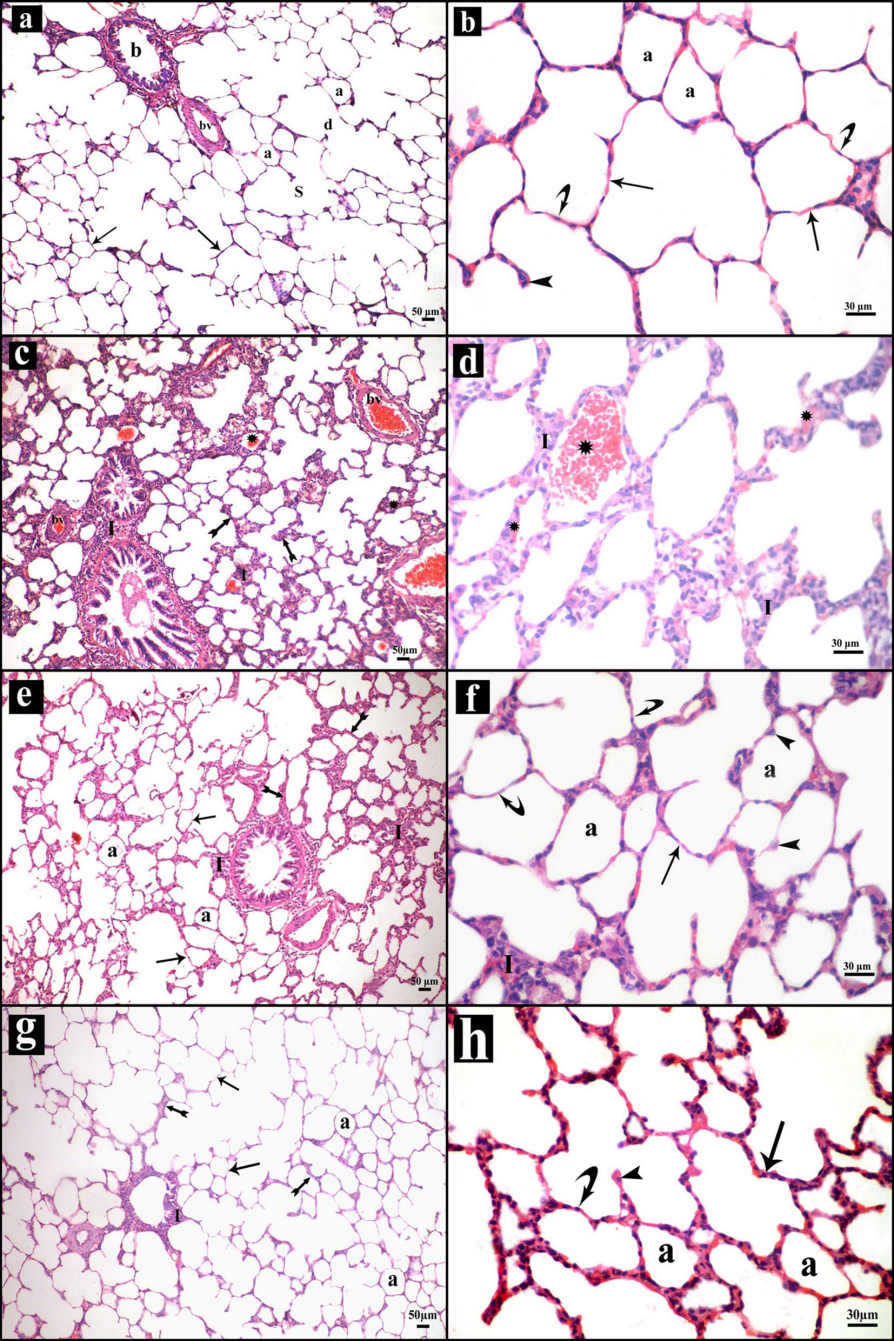
Sections stained with H&E in rats’ lung of the study groups. **(a,b)** Control group. (**a)** Normal lung structure with polygonal alveoli (a) having thin interalveolar septa (arrows), alveolar sacs (s), alveolar duct (d), bronchiole (b) and blood vessel (bv). (**b)** The lining of alveoli (a) is formed of pneumocytes type I (curved arrows) and type II (arrow heads) with thin interalveolar septa (arrows). **(c,d)** Intestinal I–R group. (**c)** Marked inflammatory cellular infiltrations (I) within and around wall of bronchioles, perivascular and within the septa that shows some thick areas (tailed arrows). Congestion of blood vessels (bv) is observed and extravasated erythrocytes (asterisk) appear in the septa and the lumen of alveoli. (**d)** Inflammatory cellular infiltrations (I), some erythrocytes (asterisk) extravasated in the septa and others in the lumen. e&f Mesna I group. (**e)** Almost normal structure of the lung with preservation of normal alveoli (a), some areas of interalveolar septa are thin (arrows), others show mild thickening (tailed arrow) with minimal inflammatory cellular infiltrations (I). (**f)** Almost all alveoli (a) are normally inflated and lined by type I (curved arrows) and type II pneumocytes (arrow heads) with thin interalveolar septa (arrows). Notice, few inflammatory cells in the interalveolar septum (I). **(g,h)** Mesna II group (**g)** nearly normal lung structure; normal alveoli (a), thin interalveolar septa (arrows) while some areas show mild thickening of the septa (tailed arrow) and few cellular infiltrations (I). (**h)** the alveoli (a) are well inflated and lined by type I (curved arrows) and type II pneumocytes (arrow heads) with thin interalveolar septa (arrows).

Sections of control group exhibited a normal structure of alveoli, alveolar sacs, alveolar ducts, bronchioles and blood vessels. Pneumocytes type I and pneumocytes type II formed alveoli lining (Fig. 2a,b).

Intestinal I–R group examination revealed marked inflammatory cell infiltrations within and around the wall of bronchioles, perivascular and within the inter-alveolar septa that lead to mild thickening in some areas. Some red blood cells were extravasated to the alveolar lumen and the interalveolar septa with blood vessel congestion (Fig. 2c,d).

Mesna I and II groups presented with a nearly normal lung structure with preservation of normally inflated alveoli with lining of pneumocytes type I and pneumocytes type II. Thin interalveolar septa with minimal inflammatory cell infiltrations and mild thickening of some areas were still detected (Fig. 2e–h).

##### Immunohistochemical results

**Figure 3:**
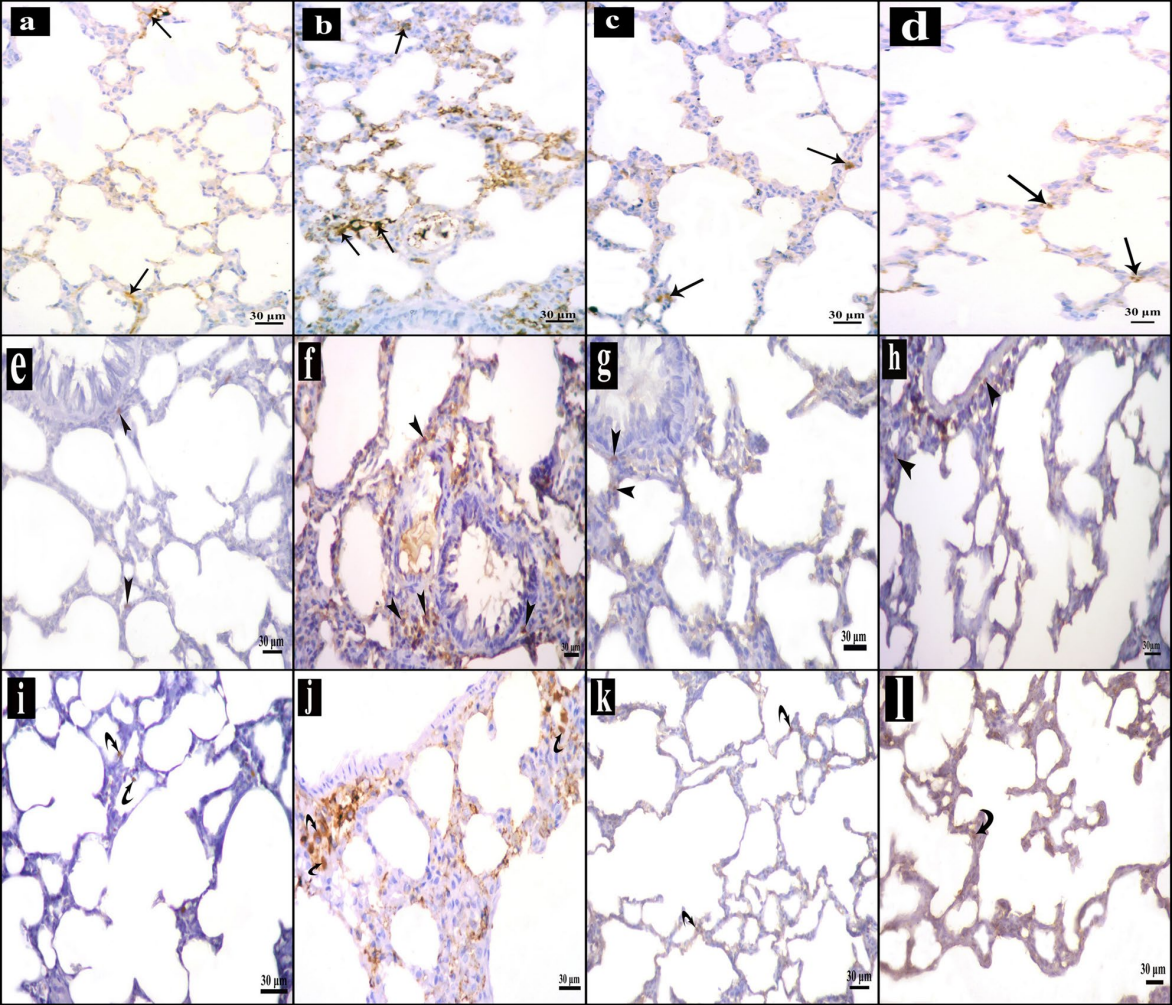
Immunohistochemically stained sections in lung of the study groups. **(a,e,i)** Control group, **(b,f,j)** intestinal I–R group and **(c,g,k)** Mesna I group. **(d,h,l)** Mesna II group. (**a–d)** Brown cytoplasmic immune reaction for P38 MAPK in the lung tissue (arrows). (**a)** Immune reactions appear in few cells. (**b)** Many immune reactive cells. (**c,d)** Reactions are still present in few cells. (**e–h)** HSP 70 immune stained sections. (**e)** Scanty positive cytoplasmic immune reactions (arrow heads) in the alveolar and bronchiolar wall. (**f)** Numerous immune stained cells. (**g,h)** The immunoreaction is decreased in comparison to that of the intestinal I–R group. (**i–l)** Brown NF-κB cytoplasmic immune-reaction (curved arrows). (**i)** The reaction appears in few cells. (**j)** Several immune stained cells. (**k,l)** Almost normal reactions with few scattered immune stained cells.

Sections stained for P38 MAPK of control group revealed brown immune reactions in the cytoplasm of few cells of the lung tissue. Intestinal I–R group revealed many reactive cells. Reaction was still present in few cells in Mesna I and II groups (Fig. 3a–d).

Stained sections of HSP70 showed scanty brown cytoplasmic immune-reactivities in the wall of alveoli and bronchioles of control group. Intestinal I–R group presented with numerous immune stained cells. Immune reactions were less in Mesna I and II groups (Fig. 3e–h).

NF-κB-stained sections of control group revealed brown immune reactions in the cytoplasm of few cells. Several immune stained cells were seen in intestinal I–R group. Normal reaction with few scattered positive cells were still present in Mesna I and II groups (Fig. 3 i–l).

#### Results of electron microscopy

**Figure 4:**
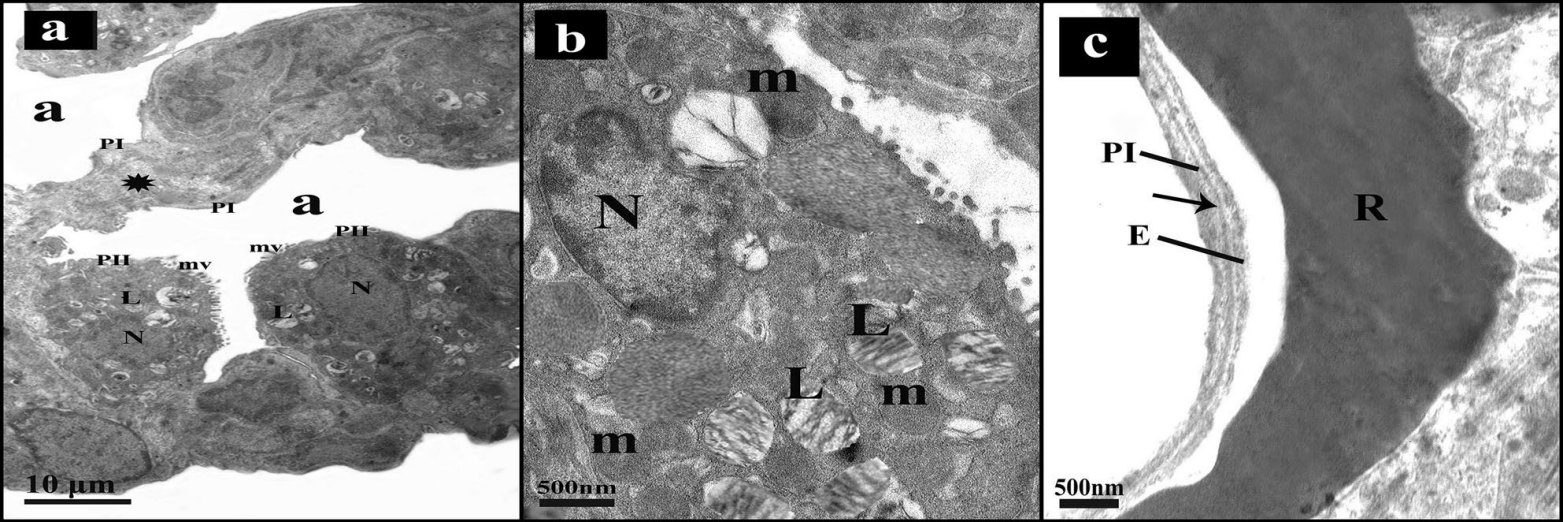
An electron micrograph of sections in lung of control group. (**a)** The alveoli (a) lining are type I pneumocytes (PI) separated by thin interalveolar septa (*) and type II pneumocytes (PII) with central regular nucleus (N), lamellar bodies (L) and short microvilli (mv). (**b)** Pneumocytes type II (PII) have central regular nucleus (N), lamellar bodies (L), mitochondria (m) and short microvilli (mv). (**c)** Blood air barrier is composed of thin cytoplasm of type I pneumocyte (PI), the fused basal laminae (arrow), and endothelial cells (E). Notice, RBCs (R) in the capillary lumen.

Control group alveoli were lined by type I and type II pneumocytes with thin interalveolar septa. Type II pneumocytes had regular central nuclei, mitochondria, lamellar bodies and short microvilli. (Fig. 4a,b). the blood air barrier was formed of attenuated pneumocyte type I cytoplasm, fused basal laminae and capillary endothelial cells (Fig. 4c).

**Figure 5:**
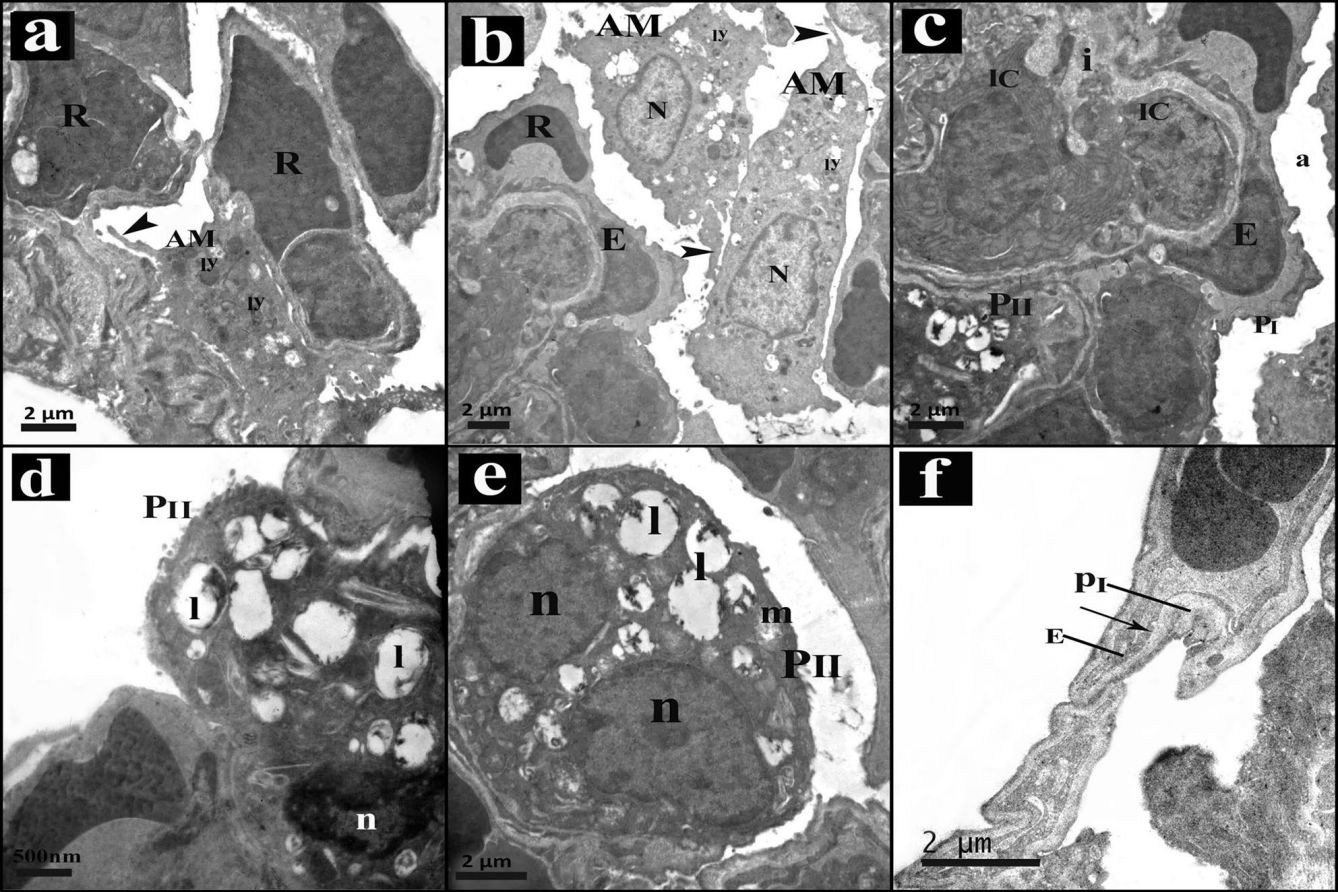
An electron micrograph of sections in lung of intestinal I–R group. (**a)** Congested capillaries with RBCs (R) are in the interalveolar septum. Within the alveolar lumen, alveolar macrophage (AM) with pseudopodia (arrow head) and lysosomes (ly) is seen. (**b)** Alveolar macrophage (AM) can be seen in the alveolar lumen, they have pseudopodia (arrow heads), heterochromatic nucleus (N) and lysosomes (ly). A capillary appears with its endothelial cell (E) and red blood cell (R) in the lumen. (**c)** Alveolar lumen (a) lining shows pneumocytes type I (PI), the capillary appears with its endothelial cell (E). The interstitium (i) is infiltrated with inflammatory cells (IC). Notice pneumocytes type II (PII). (**d)** Type II pneumocyte (PII) shows heterochromatic irregular nucleus (n) and distorted vacuolated lamellar bodies (l). (**e)** Pneumocyte type II (PII) has divided nucleus (n), some degenerated mitochondria (m) and vacuolated lamellar bodies (l). (**f)** Deformed blood air barrier composed of swollen irregular cytoplasm of type I pneumocytes (PI), fused basal lamina (arrow), and capillary endothelial cells' cytoplasm (E).

The ultrathin sections of intestinal I–R group exhibited that the blood capillaries within the interalveolar septa were congested with red blood cells. Alveolar macrophages were seen in the lumen of the alveoli with pseudopodia, heterochromatic nuclei and lysosomes in their cytoplasm. The interstitial spaces were infiltrated with inflammatory cells (Fig. 5a–c). Some pneumocytes of type II showed heterochromatic irregular nuclei with distorted vacuolated lamellar bodies, others appeared with divided nuclei and degenerated mitochondria (Fig. 5d,e). The blood air barrier appeared deformed with irregular swollen pneumocytes type I cytoplasm, fused basal lamina and endothelial cells' cytoplasm (Fig. 5f).

**Figure 6:**
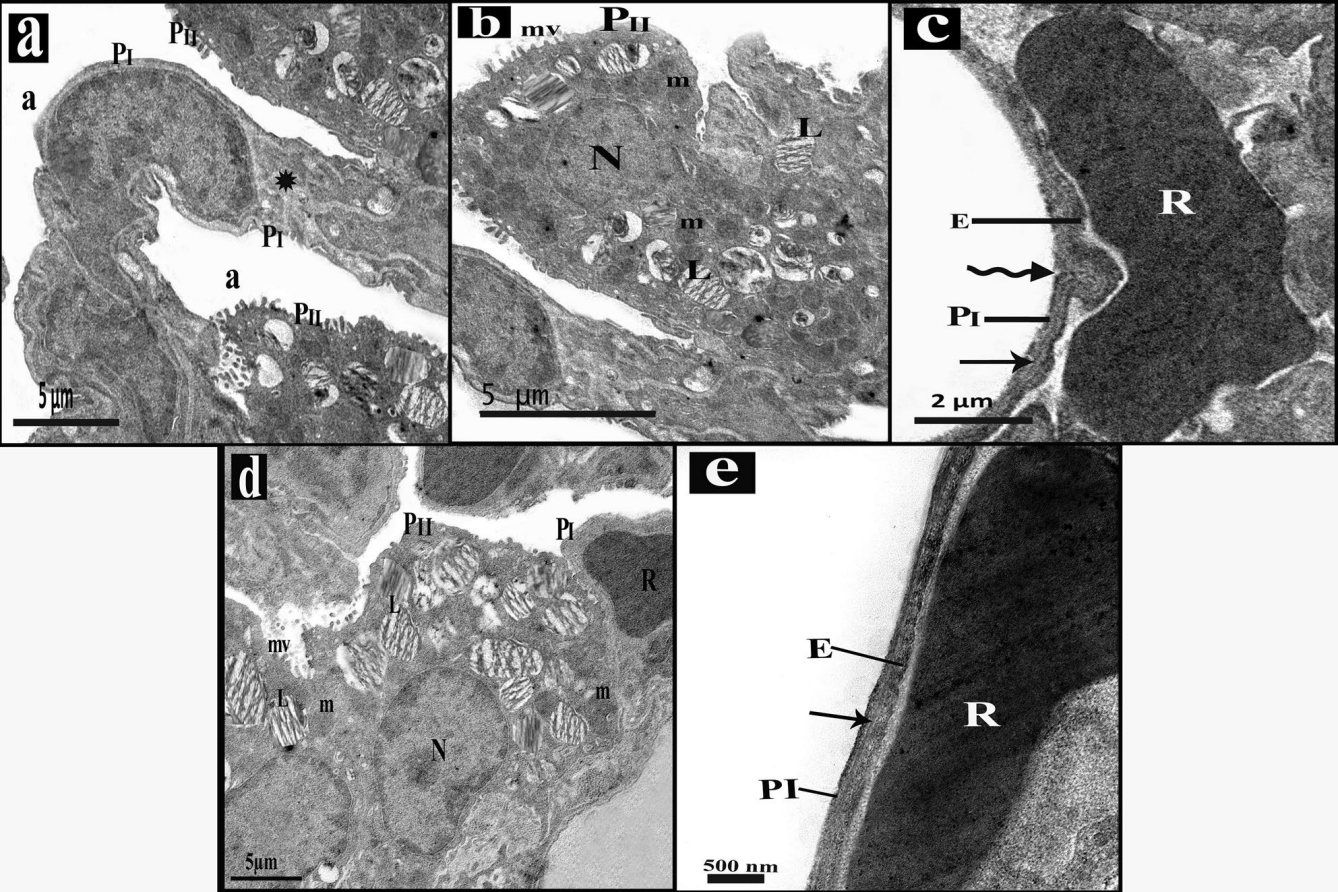
An electron micrograph of sections in lung of Mesna groups. **(a–c)** Mesna I group. (**a)** alveoli (a) lined by type I (PI) and type II (PII) pneumocytes with thin interalveolar septa (*). (**b**) Type II pneumocytes (PII) has regular nucleus (N), lamellar bodies (L), mitochondria (m) and short microvilli (mv) bulge to the lumen. (**c)** Blood air barrier appears regular except for small area (zigzag arrow). it's composed of thin cytoplasm of type I (PI), the fused basal laminae (arrow), and endothelial cells (E). Notice RBCs (R) in the capillary lumen. **(d,e)** Mesna II group. (**d)** Type I (PI) and type II (PII) pneumocytes appear lining the alveoli. Type II pneumocyte (PII) has regular nucleus (N), lamellar bodies (L), mitochondria (m) and short microvilli (mv) bulge to the lumen. notice the RBCs (R) within the lumen of the blood capillary. (**e)** blood air barrier appears regular and composed of thin cytoplasm of type I (PI), the fused basal laminae (arrow), and endothelial cells (E). Notice RBCs (R) in the capillary lumen.

Mesna I and II groups ultrathin sections examination revealed that the alveolar lining was formed of type I and type II pneumocytes with thin interalveolar septa in-between (Fig. 6a,d). Type II pneumocytes of Mesna I group showed regular nuclei, lamellar bodies, mitochondria and short microvilli bulging into the lumen (Fig. 6b). Away from small areas of irregularity in the blood air barrier in Mesna I group, it appeared regular in Mesna II group. It consisted of thin type I pneumocyte cytoplasm, fused basal laminae, and capillary endothelial cells (Fig. 6c,e).

### Morphometric results

Results for the number of p38 MAPK, HSP70 and NF-κB immune reactions and inflammatory cells' count in sections stained with H&E revealed highly significant increases in intestinal I–R group when compared to control, Mesna I and Mesna II groups. On the other hand, Mesna treatment significantly decreased the previous markers; there were non-significant differences in Mesna I group concerning the number of p38 MAPK and NF-κB immune reactions and the count of inflammatory cells when compared to the control group. Mesna II group revealed non-significant differences regarding the number of anti- HSP70, NF-κB inflammatory cells when compared to the control and Mesna I group (Table 3).

**Table 3:** Number of anti-p38 MAPK, anti- HSP70 immune-stained cells, anti- NF-κB and the number of inflammatory cells.

	Control group	Intestinal I–R group	Mesna I group	Mesna II group
Anti-p38 MAPK	2.1 ± 0.5	22.4 ± 2.2 ^##c^	6.1 ± 1.3 ^##o^	9.1 ± 1.4^#c, ##o^
Anti-HSP70	0.7 ± 0.2	37.1 ± 3.1^##c^	13.2 ± 2.0^#c, ##o^	6.9 ± 2.5^##o^
Anti-NF-κB	1.3 ± 0.4	31.5 ± 2.5^##c^	5.9 ± 1.3^##o^	4.3 ± 1.2^##o^
Inflammatory cells	2.3 ± 0.5	13.8 ± 2.4^##c^	6.7 ± 1.6^#o^	3.2 ± 0.8^##o^

Values are expressed as mean ± standard error (X ± SE).

*p38 MAPK* p38 mitogen-activated protein kinase, *HSP70* heat shock protein 70, *NF-κB* nuclear factor kappa beta.

^#^Significant difference (*P* < 0.05).

^##^Highly significant difference *(P* < 0.001); n = 10 animals.

^c^*P* compared to control group.

^o^*P* compared to intestinal I–R group.

## Discussion

Transient obliteration of the superior mesenteric artery (SMA) causes intestinal ischemia producing extensive local tissue damage. While reperfusion is required to eliminate the damage of ischemia, reperfusion itself produces more cellular damage via triggering the inflammatory response. The lung is the most vulnerable distant organ to be influenced resulting in acute systemic and lung inflammation^31–33^.

In the current study, intestinal I–R compromised the lung function as evident by hypoxemia. There was also associated metabolic acidosis. Light and electron microscopy examinations of the lung tissues revealed marked inflammatory cell infiltrations with mild thickening in some areas of the interalveolar septa. These findings were proved by morphometric analysis which displayed a highly significant elevation in the inflammatory cells count. Infiltrations were mostly neutrophils. Myeloperoxidase (MPO) was utilized to detect neutrophil sequestration. We noticed that intestinal I–R lead to a highly significant elevation in lung MPO activity. These results were aligned with those of Kim et al.^33^. Many investigators reported that I–R-induced lung injury is mainly neutrophil-dependent^34^. Neutrophil recruitment causes excessive secretion of proteolytic enzymes, like elastase and MPO^35^.

In the same respect, leukocyte-endothelial interaction exerts a pivotal role in lung injury as it could stimulate the transmigration of neutrophils and subsequently the secretion of inflammatory cytokines. ICAM-1 controls neutrophil chemotaxis, adhesion, and blood to tissues migration, resulting in organ injury and systemic inflammatory response^36,37^. In the current work, lung expression of ICAM-1 mRNA is significantly enhanced in the intestinal I–R group that was in agreement with Wang et al. results^7^. The lung inflammatory mediators; TNF-α, IL-1and IL-6 were also elevated significantly. Ramachandran et al.^38^ suggested that manufacture of inflammatory mediators like TNF-α could result from stimulation of nuclear factor-kappa beta (NF-κB) pathway contributing to exacerbation of I–R damages.

NF-κB proteins, transcription factors, influence several genes expression, which in turn control inflammatory cascades, oxidative stress responses and apoptosis^39^. We reported a highly significant up-regulation of NF-κB immune expressions. This might be attributed to the excess generation of reactive oxygen species (ROS) which activates NF-κB and stimulates its cytoplasm to nucleus translocation, where it enhances inflammatory cytokines' gene expression^40,41^.

With reference to oxidative stress responses, excessive ROS production could overwhelm the antioxidant integrity of the lung tissues causing oxidative stress^42^. In harmony with the results of Meng et al.^43^, we reported a highly significant decline in the enzymatic scavengers; GPx and SOD.

Neutrophil-produced ROS lead to distortion of endothelial barrier, increased permeability of the pulmonary vasculature and vascular leakage^44,45^. This could clarify RBCs extravasation to the lumen of alveoli and interstitial capillaries congestion described in our results. We also reported a significant increase in BAL proteins which could attributed to protein leakage secondary to altered alveolar-capillary membrane permeability. Mittal et al.^46^ stated that the loss of barrier integrity by ROS is mediated by attacking the endothelial structure, e.g. actin filament and junction composition or influencing the intercellular signaling pathways which control the barrier function.

ROS also stimulate TNF-α production that exacerbates endothelial cell injury by endorsing IL-1 and IL-6 release resulting in further neutrophil aggregation and tissue damage. Further, TNF-α induces apoptosis by stimulation of Caspase^47,48^. In the same sense, we reported up-regulation in the apoptotic marker p38 MAPK expression in the intestinal I–R group. P38 MAPK is a member of MAPKs family; a group of serine/threonine kinases which controls proliferation, differentiation, and cell survival in cells of mammals by responding to different signals e.g. growth factors and cellular stress^49^. The p38 MAPK signaling pathway stimulates apoptosis by phosphorylation of the key regulator p53^50^. Our findings were in consistence with the results of other researchers^51,52^. Additionally, p38 MAPK mediates inflammation of lung tissues following intestinal I–R by enhancement of NF-κB pathway with IL-1b release in interstitial macrophages^53,54^. P38 MAPK has been accused in apoptosis and inflammation and its stimulation worsens renal I–R^55,56^.

Heat shock proteins are a family of proteins produced following exposure to stressful circumstances e.g. heat, blood loss, hypoxia, injury by ischemia–reperfusion and other surgeries^54,57^. HSP70 maintains cellular homeostasis and directs the cellular redox status by regulating glutathione-associated enzymes^58^. In the current work, a highly significant elevation in HSP70 immune-expressions was detected. Saibil^59^ explained HSP70 cryoprotection against lung injury by its capability to bind to misfolded or damaged proteins. Mine et al.^60^ added that HSP mRNA enhanced in leucocytes resulting from intestinal I–R. Hsp70 expression was also raised following I–R in rat kidney and heart^61,62^.

In the present work, electron microscopy examination of the lung following intestinal I–R showed disrupted ultrastructure of pneumocytes type II including dark pyknotic nuclei, vacuolations, degenerated lamellar bodies and degenerated mitochondria. Hybertson et al.^63^ related these results to oxidative stress that resulted in decrease the synthesis of surfactant or destroying its components. Vacuolations and organelles swelling might develop from the disturbed membrane function with increased entrance of sodium and water. Lysosomal enzymes leakage could also aggravate organelles degeneration^64^. We also detected some pneumocytes type II with dividing nuclei. It's well defined that these cells function as progenitor cells, hence they proliferate and differentiate on demand to replace their population damaged cells and those of pneumocytes type I^65^. Existence of inflammatory cells can stimulate these cells to proliferate^66^.

Mesna is capable of concentration in several tissues owing to its small molecular size^67^. It is able to scavenge free radicals and lipid peroxidation products via its sulfhydryl group^68^. In the current work, Mesna pretreatment ameliorated intestinal I–R-induced lung changes and improved systemic oxygenation and pH. These beneficial outcomes of Mesna were built on inspection of the lung that demonstrated about normal structure apart from few mild changes. Similarly, Mesna could effectively counteract injury induced by I–R in the intestine^69^, liver^70^, spinal cord^71^, and kidney^72^.

With reference to the anti-inflammatory effects of Mesna, we detected a significant reduction in number of the inflammatory cell infiltrates in Mesna-treated groups; either before I–R (Mesna I group) or after reperfusion (Mesna II group) comparable to the intestinal I–R group. Neutrophil infiltration was also suppressed as evidenced by the highly significant reduction in the tissue MPO level. In line with our results, Mesna decreased MPO levels in kidney and liver I–R nearly to normal levels^70,72^. Jeelani et al.^73^ regarded Mesna as a powerful MPO regulator. The apparent protection of lung against inflammation resulted from the elimination neutrophil infiltration^23^.

Further, ICAM-1 mRNA lung tissue expression is significantly reduced almost to normal in Mesna-treated groups. We also detected a statistically significant decrease of the inflammatory mediators; TNF-α, IL-1β and IL-6 levels in lung tissues. Mesna also decreased total proteins in BAL. The anti- inflammatory influence of Mesna was recorded in other studies^19,20^. Hagar et al.^22^ reported that TNF-α and IL-1β levels was decreased by Mesna in rats suffering from acute pancreatitis. Even in some reports of doxrobucin treatment, Mesna could decrease levels of TNF- α following chemotherapy^74^. Triantafyllidis et al.^20^ attributed this anti-inflammatory potential of Mesna to its capability to scavenge ROS.

In the current study, the antioxidant capacity of Mesna was recognized by the significant elevation of the tissue GPx and SOD comparable to the intestinal I–R group. These findings were in line with others^67^. Mshimesh et al.^23^ stated that Mesna is capable of scavenging ROS molecules.

Both oxidative stress and inflammatory circumstances trigger the inflammatory mediator, NF-κB signaling pathway^75,76^. In the current work, a marked decline in NF-κB immune-reactions was reported in Mesna groups. Ypsilantis et al.^67^ suggested that Mesna prevents NF-κB activation by scavenging ROS through sulphydryl group binding. Triantafyllidis et al.^20^ added that Mesna decreases the nuclear translocation of NF-κΒ and inhibits its activation.

Dolgun et al.^71^ reported that Mesna reduces apoptosis (by inhibiting caspase-3 activity) following spinal cord ischemia/reperfusion injury. These data were in accordance with our findings. We revealed a highly significant decline in immune-reactions of p38 MAPK. Yeh et al.^21^ speculated that Mesna could inhibit apoptosis by preventing oxidative stress. In intestinal I–R conditions, protection of distant organs from injury is mediated through suppression of p38 MAPK which recovers the intestinal barrier^40^.

In the current work, we detected a highly significant down-regulation of HSP70 immune-expressions compared to I–R group. This might be explained by the anti-oxidative stress capacity of Mesna that consequently stabilizes the antioxidant status. Gan et al.^77^ recorded that the exogenous antioxidants intake could decrease HSP activation mediated by the stressful situations through decreasing the expression of HSP70 mRNA and other HSPs like HSP27. Alpha-tocopherol acetate decreased HSP70 mRNA levels and prevented oxidative stress^78^. Supplementation of the anti-oxidant s-allylcysteine also reduced HSP70 to almost normal level owing to the capability of s-allylcysteine to improve oxidative stress resistance of tissues and restore hepatic glutathione level^79^.

There are some limitations in the current study. In the first place, the link between Mesna and neutrophils and whether the improved outcomes are caused by neutrophil suppression need further investigations. Secondly, as only p38 MAPK pathway was used to evaluate apoptosis in the lung, further signaling pathways are required to assess the anti-apoptotic effect of Mesna.

In conclusion, Mesna attenuated the histopathological changes and apoptosis of the lung following intestinal I–R. Mesna also recovered systemic oxygenation. Mesna might promote lung protection by its anti-inflammatory and antioxidant potentials. Mesna suppressed neutrophil infiltration (as endorsed by the reduction in MPO level), reduced expression of ICAM-1 mRNA, inhibited NF-κB pathway and reduced the proinflammatory mediators; TNF-α, IL-1β and IL-6 levels in the lung tissues. Mesna maintained the antioxidant profile as evidenced by the elevation of the tissue GPx and SOD and counteracted oxidative stress as ascertained by down-regulation of HSP70 immune-expressions. Accordingly, Mesna, administered either before ischemia induction or after reperfusion, can be a promising way to counteract remote injury of the lung resulted from intestinal I–R.

## Data Availability

The data of this study are available from the corresponding author upon reasonable request.

## Abbreviations

ANOVA: One-way analysis of variance
DAB: Diaminobenzidine
ELISA: Enzyme-linked immunosorbent assay
GAPDH: Glyceraldehyde-3-phosphate dehydrogenase
GPx: Glutathione peroxidase
H&E: Hematoxylin and eosin
HSP70: Heat shock protein 70
HTAB: Hexadecyltrimethylammonium bromide buffer
ICAM-1: Intracellular adhesion molecule-1
IL-1β: Interleukin-1 beta
IL-6: Interleukin-6
I–R: Ischemia/reperfusion
MPO: Myeloperoxidase
NF-κB: Nuclear factor kappa beta
PBS: Phosphate-buffered saline
P38 MAPK: P38 mitogen-activated protein kinase
P-values: Probability values
ROS: Reactive oxygen species
RT-PCR: Reverse transcription polymerase chain reaction
SMA: Superior mesenteric artery
TNF-α: Tumor necrosis factor-alpha
UCT: Ultra-cut
